# Efficacy and safety of a novel hexaspline pulsed field ablation system in patients with paroxysmal atrial fibrillation: the *PLEASE-AF* study

**DOI:** 10.1093/europace/euae174

**Published:** 2024-06-24

**Authors:** Zulu Wang, Min Tang, Vivek Y Reddy, Huimin Chu, Xingpeng Liu, Yumei Xue, Jingfeng Wang, Jing Xu, Shaowen Liu, Wei Xu, Zhihui Zhang, Bing Han, Lang Hong, Bing Yang, Mingying Ding, Ming Liang

**Affiliations:** Department of Cardiology, General Hospital of Northern Theater Command, Wenhua Road No. 83, Shenhe District, 110016 Shenyang, China; Department of Cardiology, Fuwai Hospital of Chinese Academy of Medical Sciences, Peking Union Medical College, Beijing, China; Department of Cardiology, Helmsley Electrophysiology Center, Mount Sinai Fuster Heart Hospital, New York, NY, USA; Arrhythmia Center, Ningbo First Hospital, The First Affiliated Hospital of Ningbo University, Ningbo, China; Department of Cardiology, Beijing Chaoyang Hospital Affiliated to Capital Medical University, Beijing, China; Department of Cardiology, Guangdong Provincial People’s Hospital, Guangzhou, China; Department of Cardiology, Sun Yat-sen Memorial Hospital, Sun Yat-sen University, Guangzhou, China; Department of Cardiology, Tianjin Chest Hospital, Tianjin, China; Department of Cardiology, Shanghai First People’s Hospital, Shanghai, China; Department of Cardiology, Nanjing Drum Tower Hospital Affiliated to Nanjing University Medical School, Nanjing, China; Department of Cardiology, The Third Xiangya Hospital of Central South University, Changsha, China; Department of Cardiology, Xuzhou Central Hospital, Xuzhou, China; Department of Cardiology, Jiangxi Provincial People’s Hospital, Nanchang, China; Department of Cardiology, Shanghai East Hospital, Shanghai, China; Department of Cardiology, General Hospital of Northern Theater Command, Wenhua Road No. 83, Shenhe District, 110016 Shenyang, China; Department of Cardiology, General Hospital of Northern Theater Command, Wenhua Road No. 83, Shenhe District, 110016 Shenyang, China

**Keywords:** Catheter ablation, Pulsed field ablation, Electroporation, Atrial fibrillation, Pulmonary vein isolation, Electrophysiology

## Abstract

**Aims:**

Pulsed field ablation (PFA) is an emerging non-thermal ablative modality demonstrating considerable promise for catheter ablation of atrial fibrillation (AF). However, these PFA trials have almost universally included only Caucasian populations, with little data on its effect on other races/ethnicities. The *PLEASE-AF* trial sought to study the 12-month efficacy and the safety of a multi-electrode hexaspline PFA catheter in treating a predominantly Asian/Chinese population of patients with drug-refractory paroxysmal AF.

**Methods and results:**

Patients underwent pulmonary vein (PV) isolation (PVI) by delivering different pulse intensities at the PV ostium (1800 V) and atrium (2000 V). Acute success was defined as no PV potentials and entrance/exit conduction block of all PVs after a 20-min waiting period. Follow-up at 3, 6, and 12 months included 12-lead electrocardiogram and 24-h Holter examinations. The primary efficacy endpoint was 12-month freedom from any atrial arrhythmias lasting at least 30 s. The cohort included 143 patients from 12 hospitals treated by 28 operators: age 60.2 ± 10.0 years, 65.7% male, Asian/Chinese 100%, and left atrial diameter 36.6 ± 4.9 mm. All PVs (565/565, 100%) were successfully isolated. The total procedure, catheter dwell, total PFA application, and total fluoroscopy times were 123.5 ± 38.8 min, 63.0 ± 30.7 min, 169.7 ± 34.6 s, and 27.3 ± 10.1 min, respectively. The primary endpoint was observed in 124 of 143 patients (86.7%). One patient (0.7%) developed a small pericardial effusion 1-month post-procedure, not requiring intervention.

**Conclusion:**

The novel hexaspline PFA catheter demonstrated universal acute PVI with an excellent safety profile and promising 12-month freedom from recurrent atrial arrhythmias in an Asian/Chinese population with paroxysmal AF.

**Clinical trial registration:**

ClinicalTrials.gov Identifier: NCT05114954

## Graphical Abstract

**Graphical Abstract:**
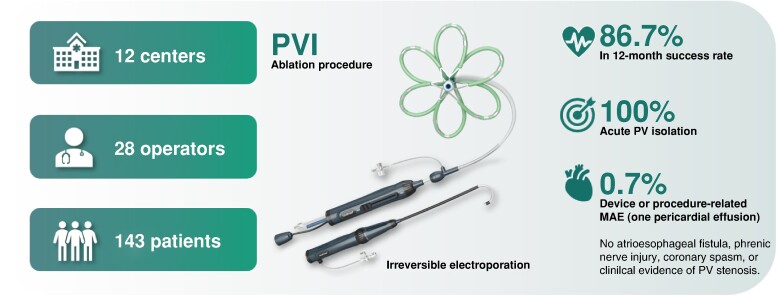

**What’s new?**

- This clinical study reports the excellent efficacy and safety of a novel hexaspline pulsed field ablation (PFA) catheter, demonstrating successful acute pulmonary vein isolation and sustained freedom from atrial arrhythmias over 12 months, with promising results for the newly designed PFA system.
- Our study supplements existing data by investigating the use of PFA in an Asian/Chinese cohort, thereby facilitating its strategic expansion into global clinical practice and promoting the adoption of this novel technology.

## Introduction

Catheter ablation is the cornerstone therapy for patients with symptomatic, drug-refractory atrial fibrillation (AF). Thermal energies including radiofrequency (RF) ablation and cryoablation have been implemented as the predominant energy sources during AF ablative treatment, but potential limitations remain—such as injury to surrounding structures (vessels, phrenic nerve, or oesophagus), pulmonary vein (PV) stenosis from post-ablation tissue proliferation, and non-durable PV isolation (PVI).^1,2^ Achieving a reduction in AF recurrence while minimizing safety complications remains a challenge for catheter ablation technologies.

Pulsed field ablation (PFA) is a largely non-thermal ablation technology with an ablative mechanism of irreversible electroporation; PFA’s unique feature is an important degree of tissue preferentiality, which results in a wide therapeutic lesion but largely sparing critical surrounding structures.^3,4^ Compared with thermal energy, PFA lesions appear wider and more clearly demarcated at a similar depth, with negligible heating and short application time. Previous published studies in small AF patient cohorts have demonstrated the feasibility of PFA for PVI, both in terms of effectiveness and safety.^5–10^ However, with only a few PFA devices available, there are currently limited data on the long-term effectiveness of PFA on a multicentre level. Additionally, potential variations in results among different ethnic groups, particularly in Asian/Chinese populations, have yet to be explored.^11^

The *PLEASE-AF* clinical trial (NCT05114954) is designed to determine the safety and efficacy of irreversible electroporation in Asian/Chinese patients with a documented history of paroxysmal AF. Pre-clinical studies with the same PFA system demonstrated effective PVI and safety, with no PV stenosis or oesophageal injury in swine compared with RF ablation.^12^ The first-in-human clinical experience involving 17 patients with paroxysmal AF also supported PFA as a safe alternative to traditional ablation methods.^13^ Here, we report the 12-month long-term clinical outcomes of using the novel hexaspline PFA catheter in the paroxysmal AF population.

## Methods

### Trial design

The *PLEASE-AF* trial was a prospective, multicentre, single-arm study conducted in China. Its purpose was to evaluate the safety and 1-year efficacy of a novel hexaspline PFA catheter in the treatment of patients with paroxysmal AF. The study began enrolment in October 2021, involving 28 operators from 12 centres in China. The study protocol was approved by the national authorities and ethics committees of each participating site. The study was funded by the manufacturer of the PFA system (CardiPulse; Hangzhou Dinova EP Technology Co., Ltd). The members of the steering committee were responsible for the scientific and clinical oversight of this study. An independent clinical events committee (CEC) assessed all adverse events.

### Study participants

Symptomatic paroxysmal AF patients (18–75 years of age), treated with at least one antiarrhythmic drug that was ineffective or intolerable, were candidates. Paroxysmal AF was defined as having AF that terminates spontaneously or with intervention within 7 days of onset, and all patients had at least one episode of AF documented by an electrocardiogram (ECG) or 24-h Holter within 12 months prior to study enrolment. Key exclusion criteria included AF secondary to reversible or non-cardiac causes, previous AF ablation or surgery, anticipated ablation other than PVI, and a diagnosis of persistent or long-standing persistent AF. All subjects provided pre-procedure written informed consent, underwent first-time PVI, and were followed for 12 months post-ablation. A pre-procedure echocardiogram was performed to confirm a left ventricular ejection fraction of ≥40% and a left atrium (LA) diameter of <5.0 cm. A transoesophageal echocardiogram was conducted to rule out the presence of atrial thrombosis. Additionally, patients underwent PV-enhanced computed tomography at baseline to identify possible future PV stenosis; however, there were no exclusions based on PV anatomy or size.

### Pulsed field ablation system

**Figure 1:**
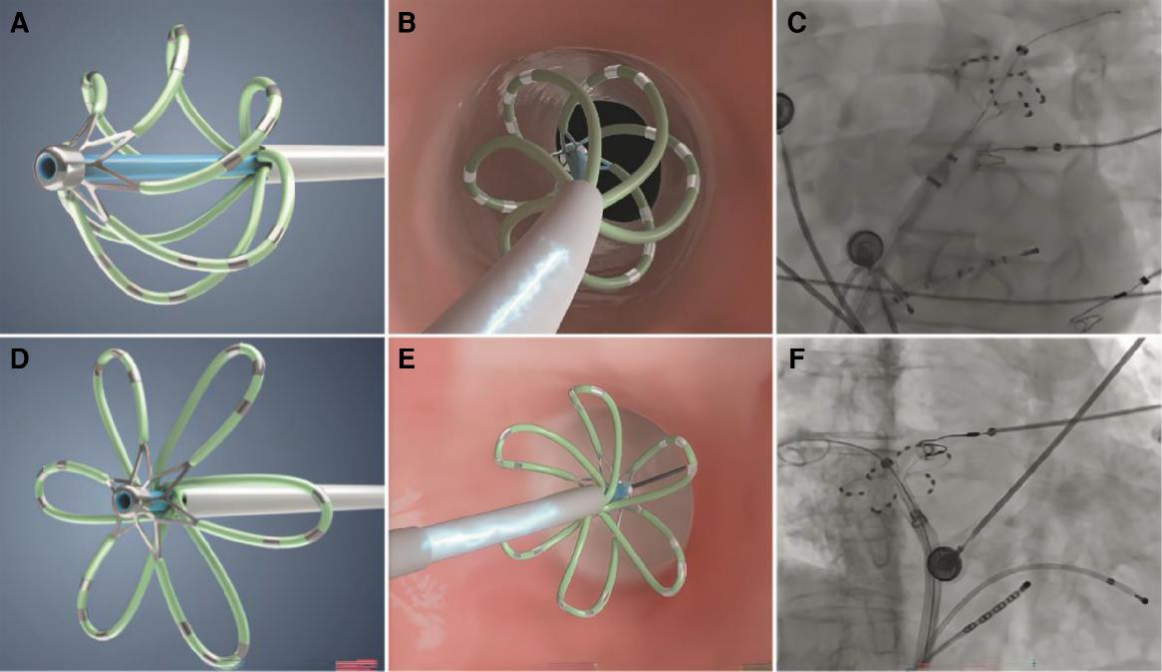
The diagram of CardiPulse catheter tip configurations. Basket configurations (*A*, *B*, *C*). Flower configurations (*D*, *E*, *F*).

The CardiPulse PFA System (Hangzhou Dinova EP Technology Co., Ltd) delivers short-duration, high-voltage bipolar biphasic pulses to a hexaspline, multi-electrode ablation catheter. The system consists of an 11F hexaspline PFA catheter, a 12F steerable sheath with a maximum 200° deflection angle capacity, and a portable touch screen pulsed field generator. The hexaspline PFA catheter includes an electrode basket, shaft, insertion tube, and handle. The electrode basket utilizes nickel–titanium alloy cutting and shaping technology, providing a framework with stronger radial support that allows the catheter electrodes to fully contact the ablation tissue. Each spline contains 3–4 electrodes, capable of simultaneously delivering biphasic pulses and recording local electrical signals. The diameter of the electrode basket is adjustable. The inner lumen of the shaft allows the injection of contrast medium and accommodates a 0.035 in guidewire to pass through, facilitating catheter positioning at the PV ostium, and the configuration of the catheter tip is adjustable from ‘basket’ to ‘flower’ configuration to accommodate different PV anatomies (*Figure 1*). The PFA catheter, available in different models, can achieve a maximal diameter of 28, 32, or 36 mm in the ‘flower’ configuration. All PFA applications were delivered in a biphasic–bipolar waveform, with sequences of microsecond scale pulses between 1600 and 2000 V.

### Procedures

Procedures were performed under either general anaesthesia or conscious sedation. Intravenous heparin was administered to obtain a target activated clotting time of ≥300 s prior to ablation and throughout the entire procedure. After routinely placing the coronary sinus and ventricular electrodes and performing a transeptal puncture, the hexaspline PFA catheter was advanced into the LA using a guidewire. Fluoroscopy or intracardiac echocardiography was used to guide PFA catheter positioning at the PV ostium, and baseline electrical potentials were recorded from all PVs. Typically, PV ablation commenced at the left superior PV (LSPV), followed by the left inferior PV (LIPV), right superior PV (RSPV), and right inferior PV (RIPV). The PFA catheter was adjusted to a ‘basket’ configuration for PV ostium ablation and then switched to a ‘flower’ configuration for PV antrum ablation (*Figure 1*). The PFA energy was delivered with different pulse intensities at the PV ostium (1800 V) and PV antrum (2000 V) for ablation, three deliveries were applied at each site, and then the catheter was rotated to a new site to ensure that any gap between electrodes was fully covered. Generally, two to three ostial sites and three antral sites were required to achieve full circumferential isolation of the PVs. This workflow was based on previous clinical work with the same system, ensuring both safety and long-term durability. During the procedure, a deflectable sheath (Hangzhou Dinova EP Technology Co., Ltd) assisted catheter manoeuvring.

Acute PVI was defined as no PV potentials within the targeted PV and entrance/exit conduction block at each PV after a 20-min waiting period. Pulmonary vein entrance block is confirmed in all patients with disappearance or dissociation of PV potentials recorded using the PFA catheter. Post-electroanatomical voltage mapping was performed at operator discretion for further confirmation of acute PVI utilizing the CARTO (Biosense Webster) or EnSite (Abbott) 3D mapping system. Administration of an isoproterenol bolus or pacing in the PV was employed in some patients at the discretion of the investigator to assess for exit block. If any residual PV connection was detected, additional lesions were placed. Additional ablation, such as superior vena cava (SVC) or others, was at physician discretion. Atrial flutter (AFL), atrial tachycardia (AT), and supraventricular arrhythmia (SVT) observed during the procedure could be ablated with commercially available ablation products—again at physician discretion. Key ablation parameters, including fluoroscopy time, total procedure time, total ablation time, ablation sites, and the total number of PFA applications, were meticulously recorded. Adverse reactions attributable to PFA, such as cough, pain, and skeletal muscle contraction, were also monitored, along with other perioperative complications. Pre- and post-procedure phrenic nerve function was evaluated in all patients by observing diaphragmatic motion during inspiration. Oesophageal temperature monitoring was not used due to the non-thermal nature of PFA.

### Follow-up

Pre-discharge transthoracic echocardiography was performed to rule out pericardial effusion. All patients returned for follow-up visits at 3, 6, and 12 months post-ablation and were assessed for arrhythmia recurrence, safety events at each visit. Recurrence of AF, AFL, or AT was evaluated by 12-lead ECG at 3-, 6-, and 12-month and 24-h Holter monitoring at 6 and 12 months. Class I or III antiarrhythmic drugs were permitted during the blanking period, but not after 90 days.

### Study endpoints

The primary efficacy endpoint was 12-month freedom from atrial arrhythmia recurrence, defined as ECG data (including surface ECG and 24-h Holter) during the efficacy evaluation period (blanking period to the end of the 12-month follow-up) without recording AF, AFL, or AT (arrhythmia monitoring device ≥ 30 s). The blanking period was 90 days following ablation. Secondary efficacy endpoints included the following: (i) the proportion of patients that achieve acute PVI, with acute PVI success referring to maintaining electrical isolation of all PVs 20 min after completing PVI; (ii) total procedure time, including the time from initial femoral venipuncture to final catheter removal; (iii) PFA catheter dwell time, defined as the time between PFA catheter insertion into the LA to withdrawal from the LA; (iv) total ablation time, defined as the accumulated time of PFA applications; and (v) total fluoroscopy time.

The primary safety endpoints for this study included the following: (i) 3-month incidence of device-related or procedure-related major adverse events (MAEs), including death, myocardial infarction, stroke or transient ischaemic attack (TIA), PV stenosis, phrenic nerve palsy, systemic embolism, pericarditis, cardiac effusion/tamponade, atrial oesophageal fistula, and severe vascular access complications; (ii) incidence of severe adverse events; and (iii) incidence of other device-related adverse events.

### Statistical analysis

Continuous data are expressed as the mean ± SD or as median (interquartile range), and categorical data are presented as number (%). Arrhythmia-free survival estimates were calculated using the Kaplan–Meier method. A *P* < 0.05 was considered statistically significant. Statistical analyses were performed with SPSS 23.0 software.

## Results

### Patient characteristics

A total of 143 patients with paroxysmal AF were enrolled between October 2021 and January 2022 at 12 centres by 28 operators, and 141 of the 143 (98.6%) patients completed the 12-month follow-up. Patients were generally young (60.2 ± 10.0 years), with more male patients (65.7%), and the most common comorbidity is hypertension (55.9%). The detailed baseline characteristics and medical history are presented in *Table 1*.

**Table 1:** Baseline patient characteristics

Characteristics	*n* = 143
Age, y	60.2 ± 10.0
Male sex	94 (65.7%)
Body mass index, kg/m^2^	25.2 ± 3.2
LVEF, %	63.4 ± 5.0
LA diameter, mm	36.6 ± 4.9
Hypertension	80 (55.9%)
Diabetes	18 (12.6%)
Coronary artery disease	17 (11.9%)
Duration of AF history, year	3.37 ± 4.76
Previous stroke or TIA in past 6m	0 (0%)
Antiarrhythmic medication	
Class I	44 (30.8%)
Class II	93 (65.0%)
Class III	31 (21.7%)
Class IV	3 (2.1%)

Numbers in the table are represented as mean ± SD or *n* (%).

AF, atrial fibrillation; LA, left atrium; LVEF, left ventricular ejection fraction; TIA, transient ischaemic attack.

### Procedural characteristics

During the procedure, 53 of the 143 patients were treated with conscious sedation, while the remaining 90 patients received general anaesthesia. No serious bradycardia requiring ventricular pacing occurred after PFA, although prophylactic atropine was typically employed. Additionally, mild to moderate coughing was occasionally observed in some patients. There was no evidence of steam pop during PFA delivery or thrombus formation on the catheter after ablation.

Procedural details are shown in *Table 2*. The total procedure time and the fluoroscopy time were 123.5 ± 38.8 min and 27.3 ± 10.1 min, respectively. The PFA catheter dwell time, which is defined as the time between the PFA catheter entering and withdrawal from the LA, was 63.0 ± 30.7 min.

**Table 2:** Procedural characteristics

Characteristics	*n* = 143
General anaesthesia	90 (62.9%)
Conscious sedation	53 (37.1%)
Number of 3D voltage mapping	71 (49.7%)
Total procedural time, min	123.5 ± 38.8
PFA catheter dwell time, min	63.0 ± 30.7
Total ablation time, sec	169.7 ± 34.6
Total fluoroscopy time, min	27.3 ± 10.1
Acute PVI success rate	143 (100%)
Non-PV ablation	10 (7.0%)
SVC	5 (3.5%)
Common-type AFL	1 (0.7%)
AT	1 (0.7%)
SVT	3 (2.1%)

Numbers in the table are represented as mean ± SD or *n* (%).

AFL, atrial flutter; AT, atrial tachycardia; PFA, pulsed field ablation; PV, pulmonary vein; PVI, pulmonary vein isolation; SVC, superior vena cava; SVT, supraventricular arrhythmia.

**Figure 2:**
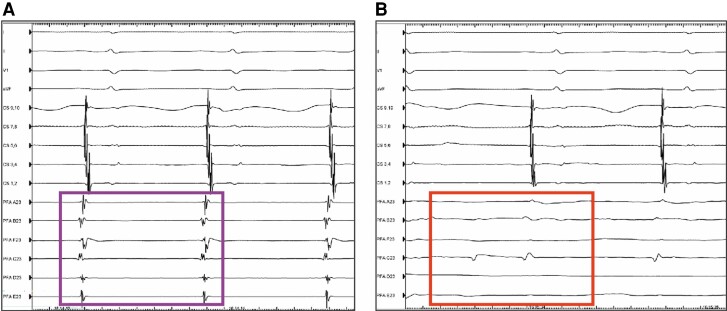
Pre- and post-ablation PV potential. After one pulsed field application, disappearance of PV potentials (*B*) compared with pre-ablation (*A*).

**Figure 3:**
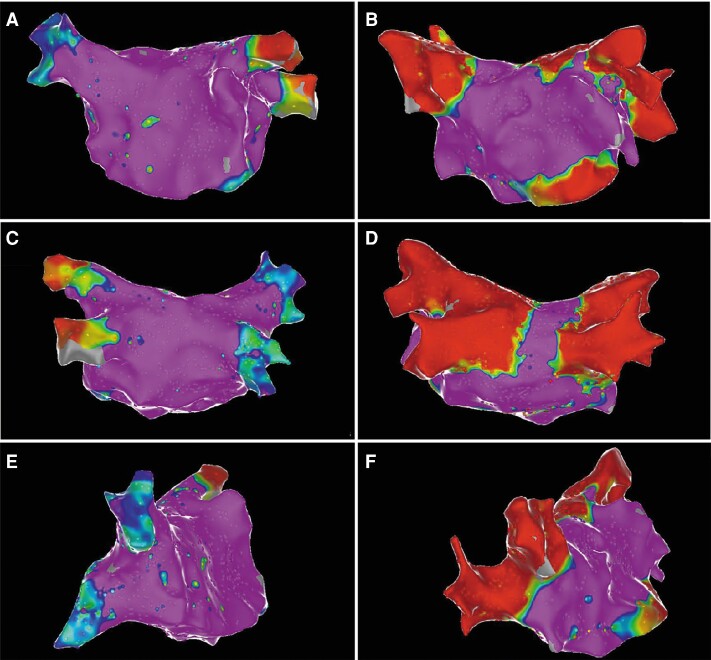
Pre- and post-ablation LA voltage maps. *A* and *B* showed antero-posterior 3D electroanatomical views of the LA pre-ablation (left panel) and post-ablation (right panel). *C* and *D* showed postero-anterior position. *E* and *F* showed right anterior oblique position. Colour purple is bipolar voltage > 0.5 mV, and colour red is bipolar voltage < 0.1 mV.

A total of 565 PVs were successfully ablated using the hexaspline PFA catheter, and acute PVI was achieved in all 565 PVs (100%), including seven common PVs. As shown in *Figure 2*, the loss of PV potential was detected in the majority of patients with the first energy application. Post-ablation voltage mapping was performed in 71 of the 143 patients (49.7%) and demonstrated complete antral PVI in all PVs without unintentional lesion formation (*Figure 3*).

In 10 out of the 143 patients, additional ablation beyond PVI was necessary during the procedure. Five patients suffered from repetitive short episodes of AF post-PVI, and the trigger origin was identified within the SVC. Subsequently, SVC isolation was performed using PFA energy with an output of 1600 V and a mean of 4.4 ± 1.5 applications. One patient developed AFL during the procedure and was ablated with irrigated RF energy across the cavotricuspid isthmus. One patient with focal atrial AT originating from the LA appendage was also ablated with RF. For the remaining three patients, paroxysmal supraventricular tachycardias (PSVTs) were diagnosed as atrioventricular re-entrant tachycardia in two and atrioventricular nodal re-entrant tachycardia in two patients. All PSVTs were successfully eliminated with RF ablation.

### Safety endpoints

One patient (0.7%) developed a pericardial effusion 1 day after the ablation procedure, which was managed conservatively without intervention; although not severe, its 2-month duration led to it being classified as a MAE. Three patients (2.1%) presented with minor vascular complications: two had groin haematomas, while one developed a pseudoaneurysm. These three patients did not receive interventional treatment or hospitalization, were categorized by the CEC as non-severe vascular complications, and therefore were not MAEs. There were no additional MAEs, either intraprocedural or during follow-up, including stroke, myocardial infarction, or death related to the PFA system. Furthermore, there were no instances of PV stenosis, stroke, TIA, or atriooesophageal fistula (*Table 3*).

**Table 3:** Safety events

Safety events	Within 3 months	4–12 months
PFA-specific adverse events		
Atriooesophageal fistula	0	0
Phrenic nerve injury/palsy	0	0
PV stenosis	0	0
Non-PFA–specific adverse events		
Systemic embolism	0	0
Pericardial effusion	1/143 (0.7%)	0
Pericardial tamponade	0	0
Stroke or TIA	0	0
Myocardial infarction	0	0
Vascular complications	3/143 (2.1%)	0
Haematoma	2	0
Arteriovenous fistula	0	0
Pseudoaneurysm	1	0
Death	0	0

Numbers in the table are represented as *n* (%).

PFA, pulsed field ablation; PV, pulmonary vein; TIA, transient ischaemic attack.

### Efficacy endpoints

**Figure 4:**
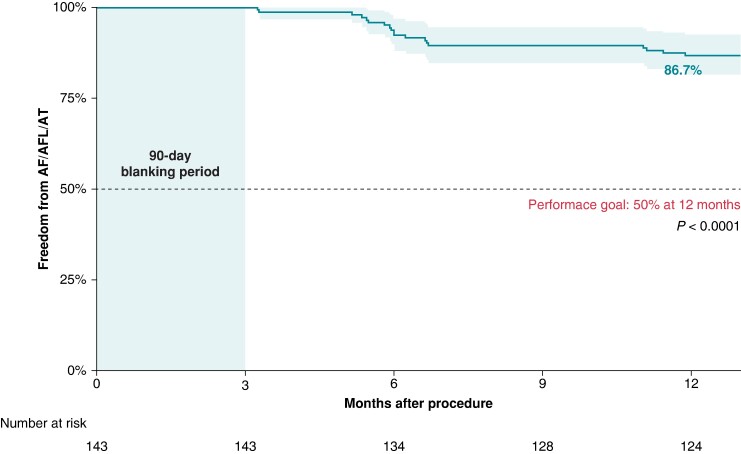
Kaplan–Meier survival curve for freedom from AF, AFL, or AT at 12 months. AF, atrial fibrillation; AFL, atrial flutter; AT, atrial tachycardia.

The majority of patients (141 of 143, 98.6%) completed the 12-month follow-up, and the primary outcome of freedom from atrial arrhythmia recurrence after the 90-day blanking period was achieved in 124 of 143 patients (86.7%; *Figure 4*). Of these, two patients were lost to follow-up, and 17 patients had recurrent atrial arrhythmias. The individual efficacy events are presented in *Table 4*. Electrocardiogram or 24-h Holter monitoring documented the recurrences as AF in 15 of 17, AFL in 1 of 17, and AT in 1 of 17 patients.

**Table 4:** Efficacy event and summary

Primary efficacy outcome	*n* = 141 of 143^a^
Freedom from AF/AFL/AT	124 of 143 (86.7%)
AF	15 of 143 (10.5%)^b^
AFL	1 of 143 (0.7%)
AT	1 of 143 (0.7%)
Lost to follow-up	2 of 143 (1.4%)
Redo ablation within 90-day blanking period	4 of 143 (2.8%)

Numbers in the table are represented as *n* (%).

AF, atrial fibrillation; AFL, atrial flutter; AT, atrial tachycardia.

^a^Two subjects were lost to follow-up during the trial, leading to incomplete data collection for the primary endpoint at 12 months.

^b^One case recurred AF and AFL simultaneously.

Four patients underwent repeat ablation within the 90-day blanking period; three of them experienced symptomatic AF recurrence, while one experienced AT. Of the 15 PVs that were remapped, 8 showed PV reconduction (LSPV 1, LIPV 2, RSPV 3, RIPV 1, and left common PV 1). All conduction gaps were successfully closed using pulsed field energy with the investigational device. Of these four redo patients, three remained in sinus rhythm at the 12-month follow-up, except for the patient with a left common PV. At baseline, Class I/III antiarrhythmic drugs were used in 75 patients. During the 90-day blanking period, Class I and III antiarrhythmic drug therapy was continued in 57 of 143 and 73 of 143 patients, respectively. After the 3-month follow-up, only five patients were still using antiarrhythmic drugs.

## Discussion

The *PLEASE-AF* clinical trial is a multicentre, prospective, single-arm study evaluating the acute clinical efficacy, periprocedural safety, and 12-month follow-up using a novel hexaspline PFA catheter in patients with paroxysmal AF. The main findings were as follows: (i) 100% acute PVI rate for all PVs (565 of 565 PVs); (ii) the primary efficacy endpoint of freedom from AF/AFL/AT was observed in 124 of 143 patients (86.7%) during the 12-month follow-up; (iii) the rate of procedure-related adverse events was low (1 of 143, 0.7%; local investigator determined); and (iv) relatively short intervention time (the PFA catheter dwell time and PFA application time were 63.0 ± 30.7 min and 169.7 ± 34.6 s, respectively).

### Efficiency

Improved efficiency was achieved in our study with a mean PFA application time of 169.7 ± 34.6 s, which is notably short compared with traditional catheter ablation and consistent with the reported PFA delivery time in other studies, which ranged from 30 to 180 s per patient.^13–15^ Additionally, we compared the PFA catheter dwell time, defined as the total time between the PFA catheter entering and withdrawing from the LA, and found a mean PFA procedure time of 63.0 ± 30.7 min. However, as operators became more experienced with PFA catheter manipulation, the overall procedural efficiency improved. It was found that the mean catheter dwell time in the late stage of this trial was reduced to 56.1 ± 16.8 min. These results also demonstrated the potential for the adoption of this hexaspline PFA catheter in daily practice.

The current PFA procedure integrates a commercial 3D electroanatomical mapping system to further confirm acute PVI. Compared with a fluoroscopy-dependent ablation strategy, the usage of a 3D mapping system helps reduce unnecessary PFA catheter dwell time or energy delivery time.^14^ In the *PLEASE-AF* study, 71 of the 143 patients underwent a 3D voltage mapping system with a AF recurrence rate of 11.3% (8 of 71 patients) at 1-year follow-up, but no difference was detected when compared with patients with a 2D mapping system (15.3%, 11 of 72 patients).

### Efficacy

Recent PFA reports have demonstrated high efficacy in the treatment of AF.^15–19^ The 1-year Kaplan–Meier estimation of freedom from atrial arrhythmia was 87.4% in the *IMPULSE* trial and 84.5% in the combined *IMPULSE*, *PEFCAT*, and *PEFCAT II* trials.^1,16^ The *MANIFEST-PF* study, a large observational registry by Turagam *et al*.^20^ (*n* = 1568), demonstrated a 1-year clinical effectiveness of 81.6% for paroxysmal and 71.5% for persistent AF. Most recently, in the randomized *ADVENT* pivotal trial of paroxysmal AF, which incorporated more intensive rhythm monitoring during follow-up, the clinical outcome in the PFA arm demonstrated a 1-year Kaplan–Meier estimation of freedom from atrial arrhythmia of 73.1%.^10^ The Kaplan–Meier estimate of AF/AT-free survival was 80% for the paroxysmal AF cohort in the real-world *EU-PORIA registry.*^21^

In *PLEASE-AF*, successful electrical PVI was achieved in all of the 565 PVs. The vast majority of the cohort (141 out of the 143 patients) completed the 12-month follow-up, and the 1-year Kaplan–Meier estimate of freedom from atrial arrhythmias was 86.7%. These data were in line with the above-mentioned findings. Of course, it should be noted that the intensity of follow-up varied between each of these various PFA trials, and for example, the first-in-human trials (*IMPULSE*, *PEFCAT*, and *PEFCAT II*) had also incorporated a ∼3-month remap procedure with re-ablation of any PV reconnections. Undoubtedly, with more consistent and rigorous monitoring (e.g. weekly transtelephonic monitoring), more arrhythmia recurrences will be captured and reflected as a reduction in long-term efficacy.^22^

Interestingly, five patients successfully underwent SVC isolation, when it was identified as a source of non-PV foci. A pre-clinical animal study in canines has been conducted to confirm the appropriate parameters for achieving SVC isolation and ensuring procedural safety of the investigational device. However, given the close proximity of the SVC to the sinus node region and considering the limited data available, it is imperative to exercise caution and prudence during related ablation procedures to prevent safety incidents. In this trial, SVC isolation was achieved in all five patients without complications such as phrenic nerve injury, SVC stenosis, or sinus node injury. Because of the relative thinness of the SVC compared with the LA wall, the PFA dose for SVC isolation was lower than that for PVI.

### Safety

The results from this trial are consistent with the safety observed in pre-clinical studies that cardiac ablation was feasible while minimizing collateral damage to neighbouring structures such as the oesophagus and phrenic nerve. The complication rate in *PLEASE-AF* was relatively low compared with recent reports, and there was no incidence of post-PFA–related atriooesophageal fistula, phrenic nerve injury, coronary spasm, or clinical evidence of PV stenosis. Although the overall safety of different PFA technologies is encouraging, collateral damage like phrenic nerve injury or coronary artery spasm has rarely been observed in real-world experience. In a retrospective survey of 24 clinical centres performing PFA in 1758 patients with AF, 0.46% experienced transient phrenic nerve paresis.^23^ These findings highlight the importance of carefully assessing phrenic nerve capture at the end of the procedure, especially for patients who are mechanically ventilated.

Additionally, coronary spasm after PFA application adjacent to coronary arteries has been reported.^24,25^ In *PLEASE-AF*, ablation was not performed near coronary arteries. Based on the potential for PFA’s proximity-related spasm, it is prudent for operators to be conscious of the possibility of spasm during ablation at the mitral or cavotricuspid isthmus.

In *PLEASE-AF*, one patient (0.7%) had a minor pericardial effusion observed soon after the procedure, but it was not haemodynamically significant and did not require intervention. Moreover, two patients with vascular haematoma and one with a pseudoaneurysm were seen, but no vascular complications led to hospitalization or intervention. It was consistent with the previously reported results.^14,19^

### Implications for pulsed field ablation treatment in Asian cohorts

The rapid evolution of PFA as a potent intervention for AF has garnered significant attention in the clinical and academic communities. The promising results from previous studies have predominantly stemmed from investigations involving Caucasian cohorts. This left an important knowledge gap regarding the application and efficacy of PFA in non-Caucasian populations, who may face economic disparities and encounter technical hurdles in accessing advanced AF treatments. Our study fills this void by being a pioneering investigation focusing exclusively on an Asian/Chinese cohort—a group previously under-represented in PFA research, thereby laying the groundwork for broader application of this technology and potential influences the clinical approach to AF management. The universal acute PVI success rate with the hexaspline PFA catheter, coupled with the favourable safety profile and 12-month freedom from recurrent atrial arrhythmias, underscores the potential of this technology in diverse populations.

## Conclusion

Pulsed field ablation using a novel hexaspline PFA catheter achieved 100% acute PVI with no incidence of post-PFA–related atriooesophageal fistula, phrenic nerve injury, coronary spasm, or clinical evidence of PV stenosis within 1-year post-procedure. The 12-month efficacy is promising, with an 86.7% freedom from any atrial arrhythmias.

## Data Availability

All relevant data are within the manuscript.
